# EEG Evaluation in a Neuropsychological Intervention Program Based on Virtual Reality in Adults with Parkinson’s Disease

**DOI:** 10.3390/bios12090751

**Published:** 2022-09-12

**Authors:** Daniela Muñoz, Patricio Barria, Carlos A. Cifuentes, Rolando Aguilar, Karim Baleta, José M. Azorín, Marcela Múnera

**Affiliations:** 1 Biomedical Engineering Department, Colombian School of Engineering Julio Garavito, Bogota 111166, Colombia; 2 Club de Leones Cruz del Sur Rehabilitation Center, Punta Arenas 6210133, Chile; 3 Electrical Engineering Deparment, University of Magallanes, Punta Arenas 6210427, Chile; 4 Systems Engineering and Automation Department, Brain-Machine Interface Systems Lab, Miguel Hernández University of Elche UMH, 03202 Elche, Spain; 5 Bristol Robotics Laboratory, University of the West of England, Bristol BS16 1QY, UK; 6 School of Engineering, Science and Technology, Universidad del Rosario, Bogotá 111711, Colombia

**Keywords:** Parkinson’s disease, virtual reality, cognitive functions, electroencephalography

## Abstract

Nowadays, several strategies for treating neuropsychologic function loss in Parkinson’s disease (PD) have been proposed, such as physical activity performance and developing games to exercise the mind. However, few studies illustrate the incidence of these therapies in neuronal activity. This work aims to study the feasibility of a virtual reality-based program oriented to the cognitive functions’ rehabilitation of PD patients. For this, the study was divided into intervention with the program, acquisition of signals, data processing, and results analysis. The alpha and beta bands’ power behavior was determined by evaluating the electroencephalography (EEG) signals obtained during the execution of control tests and games of the “Hand Physics Lab” Software, from which five games related to attention, planning, and sequencing, concentration, and coordination were taken. Results showed the characteristic performance of the cerebral bands during resting states and activity states. In addition, it was determined that the beta band increased its activity in all the cerebral lobes in all the tested games (*p*-value < 0.05). On the contrary, just one game exhibited an adequate performance of the alpha band activity of the temporal and frontal lobes (*p*-value < 0.02). Furthermore, the visual attention and the capacity to process and interpret the information given by the surroundings was favored during the execution of trials (*p*-value < 0.05); thus, the efficacy of the virtual reality program to recover cognitive functions was verified. The study highlights implementing new technologies to rehabilitate people with neurodegenerative diseases.

## 1. Introduction

Parkinson’s disease (PD) is a progressive neurodegenerative disorder that is characterized by the loss of dopaminergic neurons in the substantia nigra of the brain and, in some cases, by the presence of Lewy bodies or abnormal deposits of the alpha-synuclein protein [1]. The EP causes are mainly related to environmental factors and, in a lower percentage, by genetic factors [2]. Although the causes of the disease are not known with certainty, it is estimated that approximately, 1–3% of the elderly global population suffers from the disease, where about 200 per 100,000 inhabitants are diagnosed with PD, according to recent studies about this pathology [1,2].

The PD presents motor and non-motor symptoms where the more common are the loss of movement, muscle stiffness, and tremors at rest [3]. In general, the motor symptoms are displayed in adults older than 60 years [4], while some non-motor signs, such as the attention loss, the memory loss, the mood alteration, and the corporal pain, appear even ten years before the diagnosis of the disease. However, the non-motor symptoms that concerned the specialist the most are the neuropsychiatric ones, such as hallucinations, dementia, cognitive impairment, anxiety, and depression [5].

In the case of dementia, this pathology is associated with the presence of synucleinopathy in the cerebellar cortex and the limbic system [6]. These conditions reduce the neural capacity to receive some cerebral signals. Thus, the information transmission required for the execution of some mental functions is diminished [3]. The PD symptoms related to neuropsychologic disorders have been poorly studied. Nonetheless, some authors have demonstrated that the quality of life related to health is radically affected by these conditions, principally, because of the cognitive disability, since it makes it more difficult to perform activities such as remembering, following instructions or even can affect some movements’ speed [7].

The most common way to treat PD is the use of dopaminergic drugs that aid in controlling the symptoms through the release of dopamine [8]. As a consequence of an inexistent cure for this disease, the diagnosed people are medicated for life [8]. The use of drugs aid in the reduction of involuntary movements, but they are also provided with other treatments to diminish other symptoms such as gait disorders, reduced range of motion, memory loss, or attention deficit. Moreover, particular medicines and non-pharmacologic treatments such as cognitive behavioral treatment (CBT) are employed to treat the neuropsychologic symptoms [9].

Even though the cognitive disability is one of the more common symptoms of the PD, there are few studies regarding the strategies that may serve to rehabilitate this condition. However, some studies have revealed that the use of visual and auditive tools aid the PD patients to better concentrate and become motivated, which induces an activation of the sensory and emotional part that permit a better development of movements [10]. Most of the employed techniques in the cognitive rehabilitation are associated with the solution of mathematical problems, board games, and reading, among others. Notwithstanding, in recent years, there have been computer-assisted therapies, which, through video games such as the ERIKA platform, enable the users to exercise their mind and increase their ability to memorize and concentrate [11].

In addition to the incorporation of video games, some studies have been developed by the implementation of virtual reality (VR) in people diagnosed with PD. In one of these studies, Maggio et al. performed a qualitative evaluation of 20 patients by using the BTS Nirvana (BTS-N) system. The authors reported that after 24 sessions, the patients improved their cognitive functioning regarding the executing and visuospatial abilities, which were determined through the quality of life in the PD scales [12]. According to some researchers, the therapies based on repetitive exercises induce a positive effect on the cognitive functions; then, if the motor rehabilitation therapies are complemented with sequencing, memory, and concentration strategies, it is possible to cover as many of the PD effects as possible [13].

In the last decade, there has been an increase in the medical studies based on VR, because it has been demonstrated to improve several cortical functions and help optimize the efficiency of the sensory cortex [14], and it also makes it easier to treat patients from their home. In addition, the use of VR technology has positively impacted the memory, the daily activities, the language, the attention, the movement, and the equilibrium of the PD patients [14]. To monitor the effect of these strategies, the evaluation of electroencephalography (EEG) signals has been implemented [14,15].

A large portion of the studies performed so far with VR and EEG signals include the comparison between health and cognitive impairment-diagnosed individuals. The EEG evaluations help to detect errors in neuronal activity: for example, in the mid-frontal theta activity [15]. The development of exercises such as lifting limbs or gripping and trawling movements may have repercussions on the theta and beta bands’ power, where an increase in the potential indicates a higher activity in that specific brain region [15]. The use of games and VR have been shown to be 100% satisfactory to the participants of the studies [16,17], which may encourage adhesion to the treatments.

Although VR in the PD treatment seems to be very useful in accompanying conventional treatments, there are few studies of this technology as a strategy to improve the non-motor symptoms, such as attention, coordination, and memory loss, in which it is important to evaluate the neuronal activity of the PD after the exposure to the treatments. Given this lack of evidence about the cognitive enhancements and their relationship with the motor ones, this work seeks to study the usability of a VR-based neuropsychologic intervention program in adults with PD. The objective is to evaluate neural activity during a virtual reality-based therapy session to understand the behavior of brain activity in people with PD, once the patient is subjected to visual stimulation and when the patient is in a resting state. Therefore, this work allows understanding the influence of the therapy on attentional and memory functions as well as identifying possible deficiencies in the program protocol or corroborating the effectiveness of the intervention based on the state of the art.

## 2. Materials and Methods

**Figure 1:**
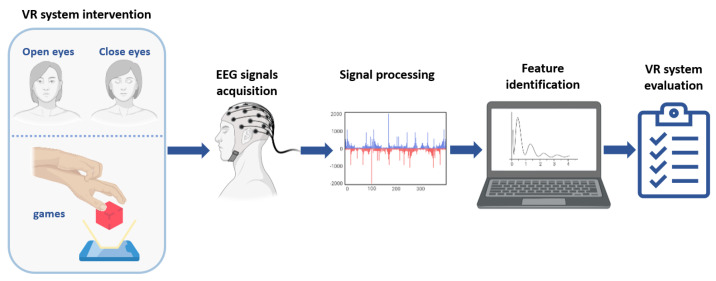
The general methodology was followed for the viability study of the VR intervention program in people diagnosed with PD.

To carry out this project, the methodology is divided into five stages related to the intervention with the VR system and the EEG signals acquisition system, the signals processing and the obtention of their characteristics, and finally, the analysis of the results. The methodology order is presented in Figure 1.

### 2.1. Intervention with the VR System

Considering that the project is focused on the study of the program’s usability, in this section, we detailed the fundamental aspects of the intervention during a session with the OCULUS QUEST-2 (Oculus VR, Irvine, CA, USA) system and the head-tracking software (Hand Physics Lab, Holonautic, Switzerland). This protocol was developed at the Corporación de Rehabilitación Club de Leones Cruz del Sur in Chile. This system aims to evaluate both the cognitive and motor functions of the participants.

#### 2.1.1. Study Population and Inclusion and Exclusion

This study involved nine patients diagnosed with PD, between 50 and 90 years of age, of both sexes; see Table 1. The sample selection was made through non-probabilistic sampling. All patients belonged to the Parkinson’s program of the rehabilitation center in Chile. The participants were diagnosed with idiopathic PD by a neurologist, classifying them as stage II and III in the Hoehn and Yahr scale. Patients with a stable dose of PD pharmacological treatment were considered. Additionally, patients able to attend and participate in the data collection process were considered. Individuals with a significant deficiency of visual or auditive functioning or with any other neurological disorder precedent were not considered in this study.

#### 2.1.2. Study Description

**Figure 2:**
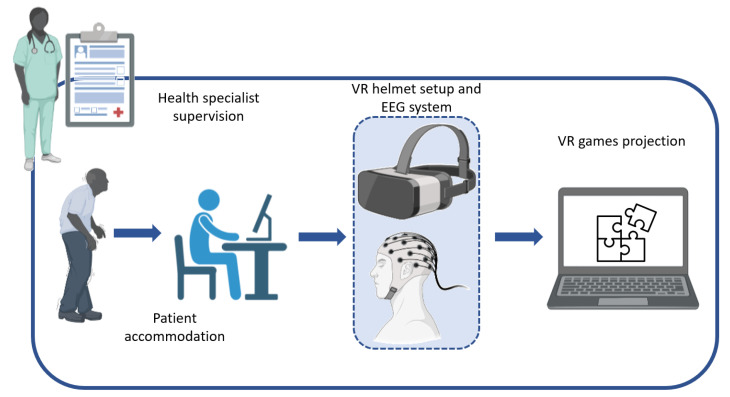
Assembly was carried out during the intervention with the VR system performed in the rehabilitation center in Chile.

The patient was placed on a chair, and the VR helmet and the EEG system ENOBIO 20 (Neuro-electrics, Barcelona, Spain) were positioned over their head. The configuration of the software was performed, specifically with the size of the 3D environment of the VR system, by the ground-level calibration. Once the equipment was ready, the treatment session began, which consisted of exercises with upper limbs and visual feedback. These activities were projected stereoscopically on VR goggles. The activities included puzzles for the treatment of cognitive and motor dysfunction. The therapist had real-time access to the activities in the 3D environment thanks to the real-time video transmission of the helmet activity (see Figure 2).

The games employed in this project were selected from the virtual laboratory Hand Physics Lab. Five games were chosen related to the ability, coordination, and memory (see Table 2). Furthermore, two control tests were performed: closed eyes (CE) and opening eyes (OE). These tests consisted of the positioning of the participants in a resting state without executing any type of activity.

### 2.2. Signals Acquisition and Processing

The data processing was performed in the software MATLAB (MathWorks, Natick, Massachusetts, USA). A Butterworth filter [18] was implemented with a lower cut-off frequency of 1 Hz and an upper cut-off frequency of 50 Hz [19], with an order of 6 to remove network noise from the EEG signals. A total of 19 signals were taken, which correspond to 19 cerebral channels (P7, P4, Cz, Pz, P3, P8, O1, O2, T8, F8, C4, F4, Fp2, Fz, C3, F3, Fp1, T7, and F7) for each of the tests performed by the patient. Each of the recordings had a duration of 3600 s with a sampling frequency of 500 Hz for all the tests performed. Once the signals were cleaned, they were visualized in the EEGLAB interface, in which it was possible to notice the presence of perturbations in some regions of the signals. The regions with more noise had a duration of about 20 s; then, by making a relationship between the signal size and the disturbance duration, it was determined that the best approach was to divide the signals into 170 windows each with a duration of about 20 s so that as much noise as possible could be removed.

After reviewing the literature, it was determined that alpha and beta were the bands that provided the most information for the analysis of functions such as memory, concentration, attention, and the ability to respond to visual stimuli. Alpha and beta bands allow us to see the influence of environmental stimuli on brain activity, with alpha being representative in the study of resting-state conditions and beta being representative of conditions of motor stimulation, so it is worthwhile to analyze these bands to understand the brain behavior of people with Parkinson’s disease [20,21,22,23]

For each of the 170 signal windows, both absolute power and relative power were calculated for the alpha and beta bands. These were calculated using the default MATLAB *Bandpower* function for the absolute power, and the relative power was obtained as the ratio of the absolute power to the segment length [20]. To this end, the signals were analyzed between 8 and 12 Hz, and 12 and 30 Hz, respectively [24]. From the powers obtained, it was determined that powers higher than 80 µV2/Hz would not be considered, because they represented noise signals. Once the signals were cleaned, the results of the segments were averaged for each channel, and the data obtained were organized in tables, in EXCEL, by cerebral lobes. In this study, a total of five regions were considered: parietal lobe, central lobe, occipital lobe, temporal lobe, and frontal lobe.

### 2.3. Statistical Tests

We performed a normality test through the Kolmogorov–Smirnov test [25], a Wilcoxon paired test to determine if the data came from the same distribution between control tests and games [26], and a Friedman test to compare the results within games and to estimate the probability that the data of each cerebral channel were equal in all the games. All the tests performed had a *p*-value < 0.05 as reference [27]. Finally, to corroborate the results obtained by the Friedman’s statistical test, a post hoc test based on the Wilcoxon test was conducted to identify the games whose results differed from the others. This test solely was made for the powers and the lobes where the Friedman’s test was statistically significant.

### 2.4. Satisfaction Survey

The satisfaction survey developed for this study consisted of 10 items related to general information of both the study and the participants. In addition, it contained 12 questions regarding the characteristics of the assembly and the intervention program’s protocol and a final question to exalt the most striking points of the study and the equipment implemented. This survey was applied to the 9 participants of the study after the VR system intervention. The questions were answered according to the level of satisfaction (very satisfied, satisfied, mostly satisfied, unsatisfied and very unsatisfied). Regarding the user’s response, a number was assigned for level of satisfaction in which five was very satisfied and one was very unsatisfied.

## 3. Results

### 3.1. Calculation of Both Cerebral Bands’ Alpha and Beta Absolute Power

First, the absolute and relative powers were obtained for both the alpha and beta bands in each game and the control tests. Table 3 shows the results of the alpha band absolute power corresponding to the OE and CE control assays, where we identified a significantly higher standard deviation, principally in the parietal, central, and occipital lobes. Furthermore, a higher activity of the alpha band in the occipital lobe was observed in both control tests. On the other hand, Table 4 presents the results of the beta band absolute power obtained in the control tests, where it was possible to determine that the beta band exhibited higher activity in the OE test when compared to the CE test.

The alpha band absolute power results obtained from the games were very scattered; see Table 5. The *Finger Painting* game exhibited significantly lower frontal and temporal lobes values than other games. In addition, the games *Sorting Cubes*, *More Switches*, and *Zero Gravity Switches* presented similar results, and regarding the control tests, they slightly increased the power of the alpha band. The *Punch the Dummy* game displayed higher values of this band than the other games.

Regarding the averages of the beta band absolute power of the games, the activity of the band increased with regard to the control tests; see Table 6.

### 3.2. Calculation of Cerebral Bands Alpha and Beta Relative Power

**Figure 3:**
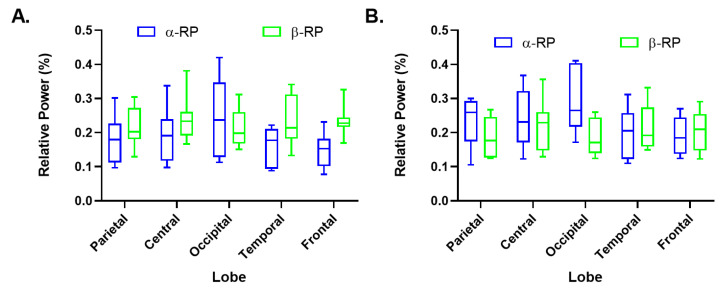
Distribution of the alpha and beta bands’ relative power by cerebral lobes of the control tests: (**A**) Opened eyes (OE). (**B**) Closed eyes (CE).

**Figure 4:**
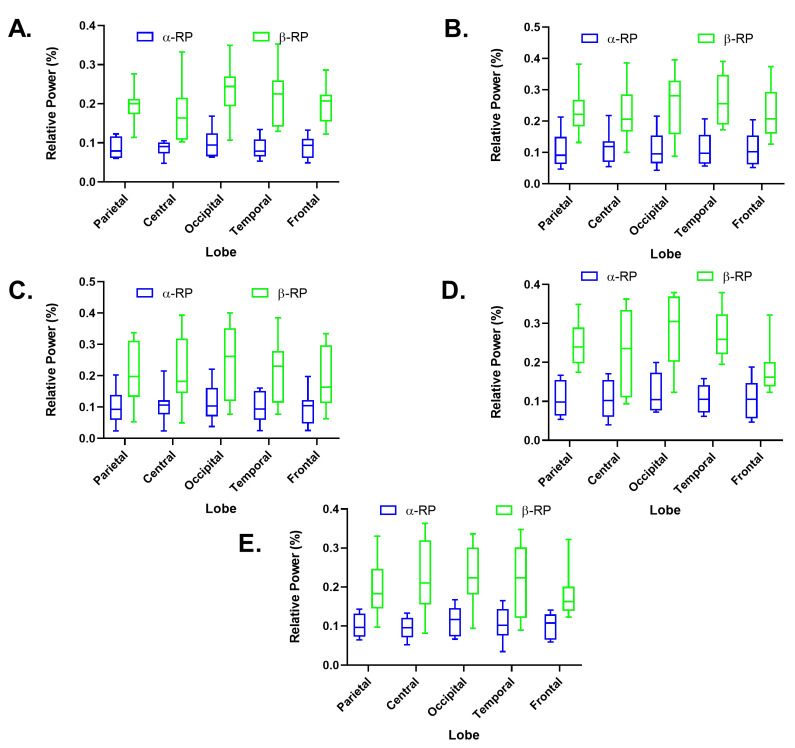
Distribution of the alpha and beta bands’ relative power by cerebral lobes of the software Hand Physics Lab games. (**A**) Finger Painting (FP). (**B**) Sorting Cubes (SC). (**C**) More Switches (MS). (**D**) Zero Gravity Switches (ZG). (**E**) and Punch the Dummy (PD).

Figure 3 and Figure 4 imaged the block diagram of both alpha and beta relative power distribution by lobes of the control tests and the games, respectively. The relative power distribution was similar between the control tests; see Figure 4. Moreover, in the OE test, we detected larger standard deviations in the alpha band relative power than in the beta band relative power; see Figure 3A. On the contrary, in both tests, we observed more homogeneous distribution of the results obtained for the beta band relative power. The higher results of the alpha band relative power were identified in the occipital and temporal regions as observed in the previously mentioned results, while the lower values were observed in the frontal areas.

In the graphs of the relative powers obtained by games, see Figure 4, it was possible to evince different values between the alpha and beta bands. The beta band relative powers reached values significantly higher. The standard deviation of the data was similar in all the games for both alpha and beta bands.

### 3.3. Normality Test and Significance of the Results

In this section, we present the relevant results concerning the statistical tests performed to all the results obtained after the processing and acquisition of the EEG signals characteristics. In the normality test of a Kolmogorov–Smirnov sample, each of the powers’ results obtained by cerebral lobes in each game and control test were considered. Then, we determined the value of h, which indicated if the null hypothesis, all the data of which came from a normal distribution, was rejected or not. For all the tests, h = 1, which was the reason why the null hypothesis was rejected.

### 3.4. Wilcoxon Statistical Test

In the Wilcoxon paired test, the results from the control tests and the games were employed. To this end, the alpha and beta bands’ absolute powers were averaged by cerebral lobes. From this test, we obtained a *p*-value for each lobe and game. In Table 7 and Table 8, we show the results of the test after comparing the alpha band absolute powers of the control tests and the games. In addition, Table 9 and Table 10 present the results of the test after comparing the beta band absolute powers of the control tests and the games. Data are only presented for the absolute power results due to the evaluation between relative powers being considered not relevant.

The results obtained for absolute alpha power with the OE test and the games, see Table 7, showed to be statistically significant in the temporal and frontal lobes only in the *Zero Gravity Switches* game, showing an increase of activity during the game in relation to the control test. Regarding the results of the test for the absolute alpha power with the CE test, see Table 8. No statistically significant results were found. This result is attributed to the similarity between the results of the tests and the deviation range of the data.

The results of the test regarding the beta band absolute power and the OE test were statistically significant in most of the cases, except for the *Finger Painting* game in the parietal, central, temporal, and frontal lobes, and the Sorting Cubes game in the parietal lobe; see Table 9. In the case of the beta band absolute power and the CE, all the games were reported as statistically significant in most of the cerebral regions, as shown in Table 10.

### 3.5. Friedman Statistical Test

To perform this test, the cerebral lobes for each game were vertically concatenated. This was completed to have each of the brain lobes of each game in the columns of the test and the participants in the rows. Table 11 displays the p-values for each of the power measures.

In the Friedman statistical test results, statistically significant results were identified, between the games, in the alpha band absolute power of the occipital, temporal, and frontal lobes and differences in the alpha and beta bands’ relative power of the central lobe between the games; see Table 11.

After the evaluation of the Friedman test results, a post hoc test was performed to corroborate the data obtained. A Wilcoxon test was performed considering the lobes and powers where the data were statistically significant. In this case, the test was executed using the alpha band absolute power of both temporal and frontal lobes and the alpha and beta bands’ relative power of the central lobe. Table 12 presents the result of this test where the games were compared one by one. In this table, it also was observed that the game Finger Painting displays more differences in the data concerning the other games.

### 3.6. Satisfaction Survey Study

**Figure 5:**
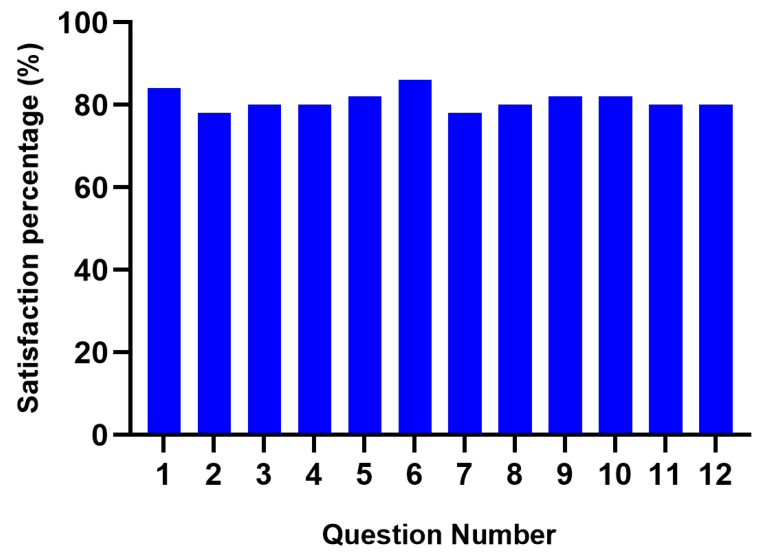
Results of the satisfaction survey displayed through satisfaction percentages according to each question performed, where 100 corresponds to “very satisfied” and 0 corresponds to “very unsatisfied”.

**Figure 6:**
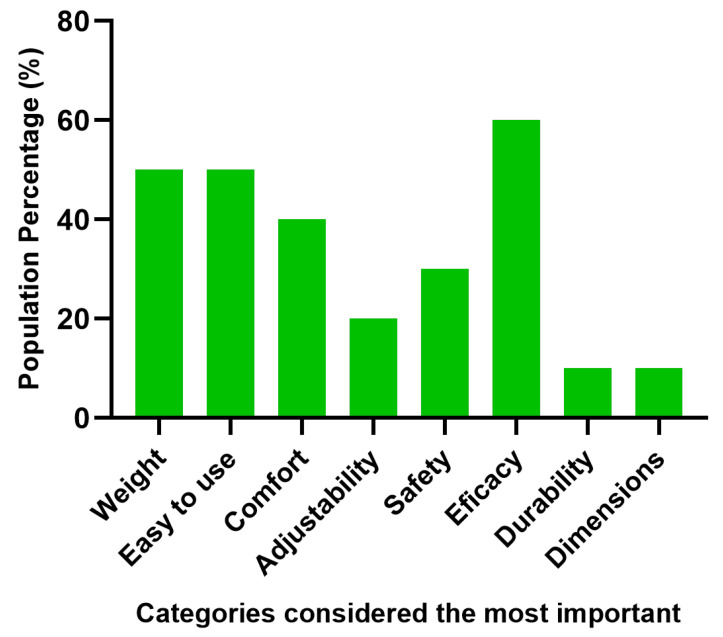
Categories considered by the users as the most important ones regarding the questions completed in the satisfaction survey.

From the information collected from the satisfaction survey, the results were organized according to the satisfaction degree. A value from 1 to 5 was assigned to each answer as follows: 1 “very unsatisfied” and 5 “very satisfied”. For each question, the answers were averaged, and the answers were graphed as a percentage of satisfaction (5 equals to 100% and one equals 0%); see Figure 5. Furthermore, the categories considered more relevant by the users were plotted next to the percentage of users that considered that category critical; see Figure 6.

The results of the statistical test evinced favorable satisfaction percentages of the study. Most of the patients remained satisfied with all the 12 parameters asked in the survey, and they expressed being very satisfied with the device and the tests performed, as shown in Figure 5.

## 4. Discussion

### 4.1. Analysis of Alpha and Beta Bands Absolute Power

According to the literature, the alpha band tends to increase its power during periods of relaxation, mainly in states where the eyes are closed [28]. This information was confirmed with the results presented in Table 3, in which there are reported higher values of the absolute power in alpha in CE than in OE. On the other hand, a study performed by R.A. Armstrong revealed visual consequences caused by PD. One of the most common conditions is the presence of visual hallucinations that increase their frequency as the disease progresses. These hallucinations are accompanied by a reduction of the ocular movement and the difficulty in differentiating visuospatial aspects. Thus, despite being under visual stimuli, the patients suffering from PD can have problems processing certain information [29]. This phenomenon may be an explanation for an increase in the alpha band activity in the occipital region of the participants during the control tests.

Regarding the beta band activity increase displayed in Table 4, the literature indicates that this band is directly related to motor and mental activities [28]. Thus, in the presence of visual stimulation, the eyes movement and the image processing increase. It was expected that during rest states, the alpha band increased its activity while the beta band’s activity decreased. However, when comparing the results of both bands in the OE test, see Table 3 and Table 4, the contrary phenomenon was observed. In their study, Bronte-Steward et al. reported an increase in the beta band activity in the PD even during relaxation periods. This feature characteristic of the disease is given by the presence of tremors, which triggers a constant motor activity [30]. Thereby, the similarity between the alpha and beta data is understood to be related to some symptoms of the disease.

By analyzing the games results, it was determined that the alpha band activity within the temporal lobe in *Finger Painting* might be associated with visual recognition; see Table 5. In the literature, it is reported that higher activity in the temporal region of the brain implies a reduction in the alpha band activity due to the rest state being interrupted [31], which supports the results obtained in our study, since the game requires attention and shapes interpretation.

The alpha band activity increased in the *Sorting Cubes*, *More Switches* and *Zero Gravity Switches* games, which may be explained by the fact that this band’s activity can increase when executing activities such as the relaxation state. This would demonstrate that the behavior of the alpha band is dependent on the attention in external and internal environments; thus, when the user is exposed to a VR-based system, they would be evaluated under an external interference-free environment, allowing a higher memory capacity, if the activities require it [32]. Nonetheless, the PD disrupted the memory action [21]. It is possible that the increase in the activity during the games’ execution is associated with the memory actions but, due to the several behaviors that this band can exhibit in the PD, it is not possible to conclude from this study the cause of the results reported.

Even if an increase in the alpha band may indicate an increase in the memory and a reduction of the band may imply an increase in the motor activity, the data from the *Punch the Dummy* game are well above the expected values according to the game description; in addition, the standard deviations are considerable, see Table 5, which does not permit theoretically analyzing the results.

As previously mentioned, the beta band tends to increase during the motor activity principally in people with proper visual imagination. Furthermore, the higher the activity of this band, the higher the visual attention [22]. The *Finger Painting* game exhibited the lowest results for the beta band absolute power; see Table 6. Then, it could be interpreted as a game that contributed the least to the increase of activity in the beta band. However, all games showed adequate activation in all the brain regions studied. Despite the standard deviations magnitude in this study, the results obtained are following the literature.

### 4.2. Analysis of Alpha and Beta Bands Relative Power

The frontal lobe oversees self-directed planning [33], which leads to the activities’ execution based on strategic learning. Given that during the control tests, the participants did not perform any activity, the low activity reported in the frontal region during the rest state is reasonable; see Figure 3.

The activity of both alpha and beta did not present a significant difference between the games; as expected, in all cases, the alpha band decreased, and the beta band increased in activity states. However, a smaller increase in the beta band was observed in the frontal region of the *Zero Gravity Switches* and the *Punch the Dummy* games (see Figure 4D,E), which is interpreted as less planning activity by the participants.

### 4.3. Analysis of Wilcoxon Statistical Test

Taking into account that the *Zero Gravity Switches* game was statistically significant for the alpha band in both frontal and temporal lobes concerning the control tests, it is possible to conclude that these differences are associated with the cognitive functions of the brain, since the behavior planning occurs in the frontal lobe [33] and the visual recognition occurs in the temporal lobe [31]. Table 3 and Table 5 display an increase in the alpha power in the *Zero Gravity Switches* game in these two cerebral lobes regarding the OE control test, leading to a low influence of the game over the cognitive functions mentioned.

Although it was expected that during the exercise, the activity in alpha decreased, it is essential to bear in mind that an increase in this band corresponds to the rise in the memory. As a result, the *Zero Gravity Switches* game is considered significant in elevating the memory capacity, and, from Table 6, an increase in the beta band can also be noted, which corresponds to the increase in visual attention motivated by this game.

For the case of the Wilcoxon test performed with the beta band absolute power data, see Table 9 and Table 10. The games significantly increased the activity of the band concerning the control tests, which supports an increase in the motor activity and principally in the visual attention as mentioned by previous authors in the literature. In addition, there was demonstrated tasks planning and visual information processing and interpretation capacity, and there was also an increase in the attention provoked by the rise in the temporal, occipital, frontal, and parietals lobes activities [31,33,34,35].

### 4.4. Analysis of the Friedman Statistical Test and the Post Hoc Test

In the Friedman statistical test, it was observed that the *Finger Painting* game has significantly different results for the alpha band absolute power regarding the other games, see Table 5, where the game exhibited lower values than the mean of the rest of the games in the temporal, occipital, and frontal lobes. This result indicates that for this game, the power reached is far below that reached by the other games, implying that it is the game that least influences the stimulation of brain activity. Other games were statistically significant for the relative powers in the Friedman test. Nonetheless, this was influenced by the polydispersity of the data, which is why the results are not considered as relevant.

Although the study sample is small, this population group allowed us to evaluate the information needed for the study. Therefore, the results obtained in the study are considered significant, considering that in other studies, the results have been promising even with a small population group [12].

### 4.5. Analysis of the Satisfaction Survey

According to the results acquired, the parameters of least satisfaction were the safety and the device comfort, which may have been influenced by the quantity of elements, such as cables and bands, that surrounded the participants’ bodies. The parameter to highlight was the facility to interact with the interface, indicating that the games of the software employed are appropriate for older adults. Finally, each of the users chose the device categories that they considered the most important, such as the efficacy of the system, the facility to use the device and its weight; see Figure 6.

Although the survey investigated the perception of the participants about the device implemented, it was not possible to obtain a qualitative evaluation of the users’ health after the sessions using the VR-based system. As a result, it was not possible to identify if the patients detected significant changes on their attention, concentration, and memory levels after conducting the tests. Unfortunately, this limits the possibility to conclude with certainty if the system positively impacts the participants’ quality of life.

## 5. Conclusions

From the study and the statistically significant results, it was possible to determine some effects of the program on the cognitive functions of the patients. It was possible to observe in the participants an increase in attention, planning, and ability to process and interpret information with most of the games used in the program. In addition, characteristics of Parkinson’s disease that influence brain activity and that differ from the normal activity of a person without neuronal pathologies were identified.

Considering the information provided in the literature on the effects of therapies based on virtual reality, it was possible to prove the action of visual stimuli over the activity of the alpha and beta bands. These bands showed the ability to maintain the cognitive functions of people during the execution of the activities. Although most of the studies found are directed to the analysis in healthy subjects, in patients with Parkinson’s disease, it was possible to observe the ability to maintain attention and satisfactorily execute each of the activities proposed for the study, regarding an increase in the activity of the beta band during the execution of the games. This allows the present work to be of interest to expand the study of VR technologies in people with PD [20,21,22,23].

Given that the results did not maintain a uniform distribution for the alpha powers, and statistically significant results were only found for this band in a single game, it is not considered relevant to determine that the VR rehabilitation program is significant for memory improvement in people with PD. After performing the study, it was possible to identify some properties of the disease that make the behavior of the alpha band differ from the behavior in people without the disease, as shown in the literature. It is expected that a second test with a larger population group and the study of signals such as EMG will allow observing wider variations and show the impact of the use of this technology in the rehabilitation of people with PD to improve different motor and non-motor functions.

Finally, the use of virtual reality technology with the games *Sorting Cubes*, *More Switches*, *Zero Gravity Switches* and *Punch the Dummy* is considered viable to improve cognitive functions related to attention, concentration, and decision making during rehabilitation therapies of Parkinson’s disease. This is concluded because increases in beta band activity were observed, which is related to an increase in activity when performing tasks involving cognitive functions, and the participants successfully executed the mentioned games. The game *Finger Painting* is not considered, because it was the game that showed less changes in the bands’ activity in relation to the other games. As observed in the results, this game was the one that least increased the activity of the bands; therefore, it is considered pertinent to study it during more sessions of the program or to test it with another game using the software.

## Figures and Tables

**Table 1:** Description of the study population.

Patients	Gender	Age	Diagnosis	Diagnostic Time
Patient 1	Male	55 years	Parkinson Desease	6 years
Patient 2	Male	61 years	Parkinson Desease	9 years
Patient 3	Male	70 years	Parkinson Desease	2 years
Patient 4	Male	64 years	Parkinson Desease	5 years
Patient 5	Male	75 years	Parkinson Desease	4 years
Patient 6	Female	71 years	Parkinson Desease	4 years
Patient 7	Female	64 years	Parkinson Desease	11 years
Patient 8	Male	64 years	Parkinson Desease	3 years
Patient 9	Female	81 years	Parkinson Desease	4 years

**Table 2:** Selected games from the software Hand Physics Lab.

Games	Characteristics
Finger Painting	Attention, Planning and Regulation
Sorting Cubes	Coordination, Attention and Memory
More Switches	Attention, Planning and Sequencing
Punch the Dummy	Coordination and Concentration
Zero Gravity Switches	Coordination, Planning and Memory

**Table 3:** Average results, by cerebral lobes, of the alpha band absolute power (µV2/Hz) obtained in the control tests opened eyes (OE) and closed eyes (CE).

Lobe	OE	CE
Parietal	13.49 ± 8.3	18.75 ± 10.22
Central	16.4 ± 11.16	21.98 ± 12.61
Occipital	23.11 ± 21.76	23.64 ± 13.13
Temporal	9.89 ± 5.45	12.68 ± 8.12
Frontal	11.85 ± 6.73	15.04 ± 7.87

**Table 4:** Average results, by cerebral lobes, of the beta band absolute power (µV2/Hz) obtained in the control tests opened eyes (OE) and closed eyes (CE).

Lobe	OE	CE
Parietal	21.05 ± 9.56	13.89 ± 5.13
Central	15.42 ± 7.85	15.8 ± 9.02
Occipital	11.95 ± 3.66	13.3 ± 5.92
Temporal	12.9 ± 6.78	10.64 ± 4.43
Frontal	15.81 ± 4.68	12.99 ± 5.01

**Table 5:** Average results by cerebral lobes of the alpha band absolute power (µV2/Hz) for the signals of the games Finger Painting (FP), Sorting Cubes (SC), More Switches (MS), Zero Gravity Switches (ZG), and Punch the Dummy (PD).

Lobe	FP	SC	MS	ZG	PD
Parietal	14.43 ± 7.88	16.08 ± 10.66	21.45 ± 9.42	18.93 ± 4.44	25.87 ± 20.65
Central	19.09 ± 18.42	18.09 ± 11.38	19.23 ± 10.69	17.25 ± 7.19	22.16 ± 21.05
Occipital	11.96 ± 5.51	22.33 ± 16.48	22.69 ± 15.57	23.38 ± 12.08	28.44 ± 15.34
Temporal	8.23 ± 2.89	14.65 ± 10.49	16 ± 11.4	16.2 ± 11.76	22.24 ± 14.21
Frontal	8.62 ± 2.57	16.22 ± 9.13	16.87 ± 6.68	17.12 ± 4.91	25.11 ± 21.88

**Table 6:** Average results by cerebral lobes of the beta band absolute power (µV2/Hz) for the signals of the games Finger Painting (FP), Sorting Cubes (SC), More Switches (MS), Zero Gravity Switches (ZG), and Punch the Dummy (PD).

Lobe	FP	SC	MS	ZG	PD
Parietal	425.24 ± 1137.14	31.71 ± 19.41	35.24 ± 10.68	40.22 ± 20.74	38.92 ± 21.72
Central	22.28 ± 10.17	30.91 ± 22.91	29.93 ± 13.27	33.37 ± 21.89	29.61 ± 24.84
Occipital	29.75 ± 20.69	34.66 ± 21.63	42.58 ± 25.57	48.99 ± 28.08	43.17 ± 27.71
Temporal	20.91 ± 12.3	28.47 ± 16.59	27.95 ± 16.79	35.61 ± 21.38	23.5 ± 6.5
Frontal	19.09 ± 9.14	29.67 ± 19.92	29.18 ± 13.61	29.74 ± 13.31	27.05 ± 19.27

**Table 7:** Wilcoxon paired test: *p*-value results of alpha band absolute power between the control test opened eyes (OE) and each of the games (Finger Painting (FP), Sorting Cubes (SC), More Switches (MS), Zero Gravity Switches (ZG), and Punch the Dummy (PD)).

Activity	Parietal Lobe	Central Lobe	Occipital Lobe	Temporal Lobe	Frontal Lobe
FP	1.00	0.74	0.74	1.00	0.74
SC	0.84	0.64	0.55	0.15	0.31
MS	0.25	0.25	0.25	0.08	0.15
ZG	0.11	0.55	0.20	**0.02**	**0.01**
PD	0.15	0.15	0.20	**0.02**	0.08

**Table 8:** Wilcoxon paired test: *p*-value results of alpha band absolute power between the control test opened eyes (CE) and each of the games (Finger Painting (FP), Sorting Cubes (SC), More Switches (MS), Zero Gravity Switches (ZG), and Punch the Dummy (PD)).

Activity	Parietal Lobe	Central Lobe	Occipital Lobe	Temporal Lobe	Frontal Lobe
FP	0.55	0.95	0.08	0.55	0.25
SC	0.64	0.84	0.64	0.64	0.64
MS	0.74	0.95	1.00	0.46	0.31
ZG	1.00	0.95	0.95	0.15	0.11
PD	0.25	0.84	0.15	**0.02**	0.15

**Table 9:** Wilcoxon paired test: *p*-value results of beta band absolute power between the control test opened eyes (OE) and each of the games (Finger Painting (FP), Sorting Cubes (SC), More Switches (MS), Zero Gravity Switches (ZG), and Punch the Dummy (PD)).

Activity	Parietal Lobe	Central Lobe	Occipital Lobe	Temporal Lobe	Frontal Lobe
FP	0.31	0.11	**0.01**	0.15	0.15
SC	0.11	**0.05**	**0.04**	**0.02**	**0.05**
MS	**0.01**	**0.01**	**0.01**	**0.01**	**0.01**
ZG	**0.02**	**0.01**	**0.01**	**0.01**	**0.01**
PD	**0.01**	**0.04**	**0.01**	**0.01**	**0.04**

**Table 10:** Wilcoxon paired test: *p*-value results of beta band absolute power between the control test opened eyes (CE) and each of the games (Finger Painting (FP), Sorting Cubes (SC), More Switches (MS), Zero Gravity Switches (ZG), and Punch the Dummy (PD)).

Activity	Parietal Lobe	Central Lobe	Occipital Lobe	Temporal Lobe	Frontal Lobe
FP	**0.04**	**0.02**	**0.02**	0.08	0.08
SC	**0.04**	**0.05**	**0.01**	**0.04**	**0.04**
MS	**0.01**	**0.02**	**0.02**	**0.02**	**0.02**
ZG	**0.01**	**0.02**	**0.01**	**0.02**	**0.02**
PD	**0.01**	**0.04**	**0.01**	**0.01**	**0.01**

**Table 11:** Friedman test. Results of the *p*-value between all the games by cerebral lobes. *P*-values below 0.05 are considered statistically significant.

Measure	Parietal Lobe	Central Lobe	Occipital Lobe	Temporal Lobe	Frontal Lobe
Alpha absolute power	0.22	0.99	**0.05**	**0.03**	**0.01**
Beta absolute power	0.31	0.69	0.23	0.21	0.52
Alpha relative power	0.90	**0.05**	0.76	0.78	0.18
Beta relative power	0.23	**0.05**	0.12	0.15	0.34

**Table 12:** Wilcoxon test results for statistically significant data from the Friedman test. Comparison between the games (Finger Painting (FP), Sorting Cubes (SC), More Switches (MS), Zero Gravity Switches (ZG), and Punch the Dummy (PD)).

Game Comparison	Alpha Absolute Power—Temporal Lobe	Alpha Absolute Power—Frontal Lobe	Beta Relative Power—Central Lobe	Alpha Relative Power—Central Lobe	Alpha Relative Power—Central Lobe
FP-SC	0.11	**0.04**	0.20	**0.02**	0.08
FP-MS	**0.02**	**0.02**	**0.02**	**0.01**	**0.01**
FP-ZG	**0.02**	**0.01**	0.11	0.38	**0.01**
FP-PD	**0.01**	**0.01**	0.38	0.08	**0.01**
SC-MS	0.38	0.95	0.25	**0.02**	0.55
SC-ZG	0.38	0.55	0.84	0.95	0.74
SC-PD	0.11	0.25	0.11	0.11	0.31
MS-ZG	0.55	0.95	0.84	**0.04**	1.00
MS-PD	0.15	0.25	**0.01**	**0.01**	0.55
ZG-PD	0.15	0.55	0.08	0.55	0.20

## Data Availability

The datasets presented in this study can be found online at: https://figshare.com/articles/dataset/EEG_PD_DATA_zip/19938446. Access date [31 May 2022]. https://doi.org/10.6084/m9.figshare.19940678. Access date [31 May 2022].
